# Relative roles of TGF-β1 and Wnt in the systemic regulation and aging of satellite cell responses

**DOI:** 10.1111/j.1474-9726.2009.00517.x

**Published:** 2009-12

**Authors:** Morgan E Carlson, Michael J Conboy, Michael Hsu, Laurel Barchas, Jaemin Jeong, Anshu Agrawal, Amanda J Mikels, Smita Agrawal, David V Schaffer, Irina M Conboy

**Affiliations:** 1 Department of Bioengineering, University of California BerkeleyBerkeley, CA, USA; 2 Division of Basic and Clinical Immunology, University of CaliforniaIrvine, CA, USA; 3 Department of Developmental Biology, Stanford UniversityStanford, CA, USA; 4 Department of Chemical Engineering and The Helen Wills Neuroscience Institute, University of CaliforniaBerkeley, CA, USA

**Keywords:** aging, anti-aging, cytokines, skeletal muscle

## Abstract

Muscle stem (satellite) cells are relatively resistant to cell-autonomous aging. Instead, their endogenous signaling profile and regenerative capacity is strongly influenced by the aged P-Smad3, differentiated niche, and by the aged circulation. With respect to muscle fibers, we previously established that a shift from active Notch to excessive transforming growth factor-beta (TGF-β) induces CDK inhibitors in satellite cells, thereby interfering with productive myogenic responses. In contrast, the *systemic* inhibitor of muscle repair, elevated in old sera, was suggested to be Wnt. Here, we examined the age-dependent myogenic activity of sera TGF-β1, and its potential cross-talk with systemic Wnt. We found that sera TGF-β1 becomes elevated within aged humans and mice, while systemic Wnt remained undetectable in these species. Wnt also failed to inhibit satellite cell myogenicity, while TGF-β1 suppressed regenerative potential in a biphasic fashion. Intriguingly, young levels of TGF-β1 were inhibitory and young sera suppressed myogenesis if TGF-β1 was activated. Our data suggest that platelet-derived sera TGF-β1 levels, or endocrine TGF-β1 levels, do not explain the age-dependent inhibition of muscle regeneration by this cytokine. *In vivo*, TGF-β neutralizing antibody, or a soluble decoy, failed to reduce systemic TGF-β1 and rescue myogenesis in old mice. However, muscle regeneration was improved by the systemic delivery of a TGF-β receptor kinase inhibitor, which attenuated TGF-β signaling in skeletal muscle. Summarily, these findings argue against the endocrine path of a TGF-β1-dependent block on muscle regeneration, identify physiological modalities of age-imposed changes in TGF-β1, and introduce new therapeutic strategies for the broad restoration of aged organ repair.

## Introduction

Adult skeletal muscle robustly regenerates throughout an organism’s life but, with advancing age, its ability to repair diminishes and ultimately fails (Grounds, 1998; Renault *et al.*, 2002). Muscle satellite cells play a crucial role in this regenerative process, and are physically situated beneath the basal lamina of myofibers (i.e. differentiated muscle cells) (Mauro, 1961). Upon myofiber damage, quiescent satellite cells become activated and begin proliferating as myogenic progenitor cells, which later differentiate into fusion-competent myoblasts and *de novo* multinucleated myofibers (Collins *et al.*, 2005; Wagers & Conboy, 2005). Although diminished satellite cell numbers have been implicated in the age-related decline of muscle tissue regeneration, several reports identified key molecular changes within Notch and P-Smad3 signaling in the aged endogenous populations (Conboy *et al.*, 2003; Carlson & Conboy, 2007b; Carlson *et al.*, 2008). In this regard, aging of the satellite cell micro-niche was found to promote underproduction of Notch ligand Delta, and overproduction of transforming growth factor-beta (TGF-β). Consequently, satellite cells residing in old muscle fibers express excessive levels of CDK inhibitors, and thus fail to break quiescence and proliferate in response to injury. However, the regenerative responses of aged satellite cells can be rescued, either by forced activation of Notch, or by shRNA targeting of Smad3 (Schultz & Lipton, 1982; Conboy *et al.*, 2003; Carlson *et al.*, 2008). The regenerative capacity of aged muscle can also be enhanced by exposing old satellite cells to young systemic factors – such as when young and old mice are physically connected to share blood circulation (parabiosis), or when aged satellite cells are cultured in the presence of young sera (Conboy *et al.*, 2005; Carlson & Conboy, 2007a). This improved regeneration of aged muscle is due to the enhanced activity of endogenous aged satellite cells, and not from physical contributions of young cells within the common circulation (Conboy *et al.*, 2005). Therefore, in addition to local tissue niches, systemic factors in sera also regulate the regenerative responses of organ stem cells and importantly, the aged circulation *inhibits* the regenerative potential of even young satellite cells (Carlson & Faulkner, 1989; Brack & Rando, 2007; Carlson & Conboy, 2007a). This evidence suggests, in the case of heterochronic parabiosis, the inhibitory factors introduced into shared circulation by old partners were continuously removed, or functionally neutralized, by the young partners.

It was reported that the aged circulation inhibits satellite cell responses by acting through the Wnt pathway (Brack *et al.*, 2007). These findings raised particular interest, as Wnt proteins are not known to be endocrine in nature, and Wnt signaling was recently demonstrated to enhance myogenic proliferation and differentiation in both embryonic and adult development (Ikeya & Takada, 1998; Marom *et al.*, 1999; Wagner *et al.*, 2000; Shi *et al.*, 2002; Brack *et al.*, 2008; Le Grand *et al.*, 2009). A recent study from our laboratory implicated the TGF-β family in the local inhibition of aged satellite cell responses (Carlson *et al.*, 2008). We therefore decided to explore a potential age-dependent endocrine activity of TGF-β, and its potential cross-talk with Wnt. Moreover, we extrapolated our previous findings by narrowing down the anti-myogenic inhibitory activity to one TGF-β family member, namely TGF-β1 (Li *et al.*, 2004; Shen *et al.*, 2008).

Transforming growth factor-beta proteins are multifunctional cytokines, secreted by numerous cell types. They are capable of signaling to virtually every cell type and broadly control cell proliferation, differentiation, apoptosis, inflammation and scarring in various tissues (Massague, 1998; Massague & Chen, 2000; Derynck & Zhang, 2003). TGF-β ligands are synthesized by cells and secreted as precursor complexes that are composed of mature dimeric C-terminal polypeptides; specifically, the latency associated propeptide and the latent TGF-β binding protein (LTBP) (Annes *et al.*, 2003; Hyytiainen *et al.*, 2004). These complexes can be transported in the blood, and are normally targeted to tissue extracellular matrix by the appropriate LTBP, where the mature polypeptide is cleaved. These activated ligands are then capable of binding to their specific receptors, thereby initiating TGF-β/P-Smad signal transduction. Several validated small molecule inhibitors, which specifically interfere with TGF-β receptor I kinase activity, are capable of blocking Smad phosphorylation upon receptor I ligand binding (Singh *et al.*, 2003; Gellibert *et al.*, 2004). Similarly, an exogenous dominant-negative form of TGF-β receptor II has been shown to effectively prevent TGF-β signaling in several cell types (Brand *et al.*, 1993; Tang *et al.*, 1999). Both active and inactive TGF-β proteins are found in sera (Assoian *et al.*, 1983; Massague, 1987), whereby inactive TGF-β, transported in blood plasma, is available for activation locally in tissues. In this regard, blood cells (mostly platelets and CD4^+^ T lymphocytes) are known to produce and release high levels of total and biologically-active TGF-β ligands (Assoian *et al.*, 1983). Considering the strong morphogenic properties of TGF-β, changes in the levels of either total (latent plus active) or active form alone, are likely to influence immune responses, tissue maintenance and regeneration.

The results shown here argue against the notion of systemic TGF-β1 endocrine activity and strongly suggest that TGF-β, released by the known process of platelet activation during sera collection, inhibits satellite cell responses *in vitro.* These findings also suggest that young sera may contain a functional and natural decoy of TGF-β1, or a competitor of TGF-β1 signaling pathway (either endocrine or released by platelets). Lastly, our results demonstrate that Wnt antagonizes, rather than synergizes with TGF-β1-mediated satellite cell response inhibition.

## Results

### Defining the inhibitory range of systemic TGF-β1

The TGF-β family is composed of roughly 35 different ligands. In addition to other cytokines (e.g. IGF-1, TNF-α, IL-6, etc.) many of these have been implicated in the process of aging, and with respect to muscle regeneration (Grounds, 2002; Moresi *et al.*, 2008; Dorshkind *et al.*, 2009). Here, we focused on first determining whether the aged circulation is inhibitory to adult myogenesis through the activity of one particular family member – TGF-β1.

**Fig. 1:**
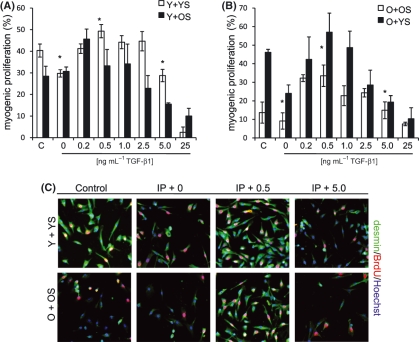
Old sera inhibits satellite cell responses by transforming growth factor (TGF)-β-dependent mechanism. Young (A) and old (B) myofiber-associated myogenic progenitor cells were isolated 3 days postinjury and cultured overnight in Opti-MEM containing either 10% young serum (YS), 10% old (OS), TGF-β1 antibody depleted serum alone, or with fixed amounts of recombinant TGF-β1 in the culture system. Cells were cultured with their specific sera for 24 h, and transferred to differentiation medium for additional 48 h (Fig S2). BrdU was added for the last 2 h to measure proliferation. Cells were then fixed and immunostained for desmin (green) and BrdU (red), with Hoechst (blue) marking all nuclei (as shown in C), and scored in multiple random fields from the above assays. Results are displayed as the mean percent of Desmin^+^/BrdU^+^/total cells, ±SD. *P < 0.05 for isochronic Y+YS/O+OS 0 ng mL^−1^, compared to 0.5 ng mL^−1^, and 5.0 ng mL^−1^ compared to 0.5 ng mL^−1^; *n* = 3.

**Fig. 3:**
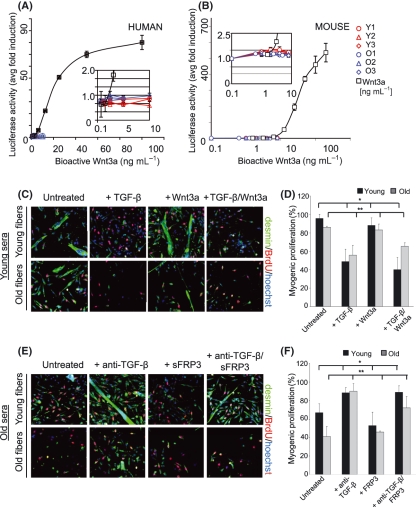
Old serum inhibits satellite cell responses by a Wnt-independent mechanism. Bioactive human (A) and mouse (B) Wnt is undetectable in human or mouse sera, and is not elevated with age. Human serum was collected from three young (∼20–25 years) and three old (∼65–75 years) individuals. Mouse serum was collected from four young and four aged mice. Levels of biologically active Wnt were analyzed, using a Wnt-reporter expressing cell line. As compared to recombinant Wnt3A, no detectable Wnt activity was found in either young or old serum. Raw fluorescence values for young sera were marginally higher compared to old sera, but both were not significantly different from the negative control (no Wnt3a samples). Inset panel shows 0.1–10 ng mL^−1^ Wnt3a range in greater detail. Myofiber-associated myogenic progenitor cells were isolated 3 days postinjury and cultured overnight in Opti-MEM containing either (C) 10% young serum (YS); YS+Wnt3A; YS+Wnt3A+TGF-β; or (E) 10% old (OS), OS+FRP3, OS+FRP3 + anti-TGF-β. BrdU was added for the last 2 h to measure proliferation. Cells were then fixed and immunostained for desmin (green) and BrdU (red), with Hoechst (blue) marking all nuclei. TGF-β addition reduced myoblast and myotube production in young serum, TGF-β neutralization resulted in a much improved myogenic proliferation in old serum, and some cells even formed *de novo* myotubes. In contrast, exogenous Wnt3A did not decrease myogenic responses in young serum and FRP3 did not rescue myogenic responses in old serum. No synergy in regulation of myogenesis was detected between Wnt and TGF-β. (D) Quantification of C. Cells were scored in multiple random fields from the above assays and the results displayed as the mean percent of BrdU^+^, desmin^+^/total cells, ±SD. *P < 0.05 between young untreated or +Wnt3a vs. +TGF-β or +TGF-β/Wnt3a; **P < 0.05 for old fibers, as described above for young. *n* = 3 for each set. (F) Quantification of E. Cells were scored and displayed as in E. *P < 0.05 between young fibers + OS untreated or +FRP3 vs. +anti-TGF-β or +anti-TGF-β/FRP3; **P < 0.05 for old fibers as described for young fibers; *n* = 3 for each set.

We specifically postulated that TGF-β1 levels of young sera are benign or positive to myogenic regulation, while TGF-β1 levels found in aged circulation are inhibitory. To explore this, we first depleted TGF-β1 from young and old mouse serum, followed by re-addition at a range of tested concentrations. Muscle stem cells, derived from young and old mice, were then cultured in the presence of young and old TGF-β1-depleted, or modified, mouse serum. Τhe regenerative responses of these cells were assayed, *in vitro.* Sera was depleted of TGF-β1 by incubation with a TGF-β1-specific antibody (or isotype-matched control IgG), followed by removal of the TGF-β1–antibody complexes (or control IgG antibody complexes) using protein G-coated agarose beads. The success of TGF-β1 depletion was also confirmed by ELISA (not shown). Muscle stem cell myogenic regenerative potential was quantified, based on their ability to generate *de-novo* myogenic lineages – i.e. BrdU-incorporating desmin^+^ myoblasts (*myogenic proliferation*) that differentiate into postmitotic eMyHC^+^ myotubes (*myogenic differentiation*) (Conboy *et al.*, 2003, 2005; Sherwood *et al.*, 2004). The time course of such *de-novo in vitro* myogenesis recapitulates *in vivo* muscle repair (Conboy & Rando, 2002; Conboy *et al.*, 2005), and is shown in Supporting Fig. S1A–D. In agreement with previous findings (Carlson & Conboy, 2007a), myogenic proliferation (24–48 h of culture) and myogenic differentiation (48–72 h of culture) were consistently higher for young satellite cells and in young serum at all time points, compared to old (Figs 1 and S1A–D). Nonmyogenic, desmin^−^ proliferating fibroblasts were less numerous in cultures with experimentally calibrated levels of TGF-β1 (Fig. 1), and also declined in cultures with exogenous Wnt3A addition (Fig. 3, below). These cells are known to contaminate satellite cell preparations at ∼2–5%, and to expand in the presence of old serum or high levels of TGF-β (Carlson *et al.*, 2008).

Very interestingly, the productive myogenic proliferation of young and old muscle stem cells was robust in TGF-β1-depleted serum, *only* when low levels of recombinant TGF-β1 were introduced (Fig. 1A–C). At 1–5 ng mL^−1^ (and higher), TGF-β1 alone sufficed for the inhibition of satellite cell responses, while myogenesis was positively regulated at 0.2 ng mL^−1^ (Fig. 1A–C). Similarly, myogenic differentiation responses from young cells also peaked in TGF-β1-depleted serum, which received low levels of exogenous recombinant TGF-β1 (Supporting Fig. S2A). In contrast, old cell differentiation was improved by TGF-β1 depletion from serum alone, as well as in a low range of recombinant TGF-β1 addition (Supporting Fig. S2B). The overall differentiation response from old cells was also diminished, compared to young cells (Supporting Fig. S2A,B). As myogenic differentiation was assayed at 48–72 h of culture, and aged satellite cells have elevated TGF-β1 production (Carlson *et al.*, 2008), these data suggest that endogenous TGF-β1 production by old cells, *in vitro,* counteracts the pro-myogenic effect of TGF-β1 depletion from mouse serum (Figs 1 S1A–D and S2A,B).

Together, these data demonstrate that sera-derived TGF-β1 inhibits satellite cell responses, and that specific levels of TGF-β1 are required for productive myogenic responses. Even with complete TGF-β1 depletion, old serum remained less myogenic than young (by ∼10–20%), suggesting that while TGF-β1 is a main inhibitor of satellite cell responses, it is not the only suppressor of regeneration present in old sera (Figs 1A–C, S2A,B).

### Correlating the inhibitory range of TGF-β1 with physiological levels found in young and old sera

**Fig. 2:**
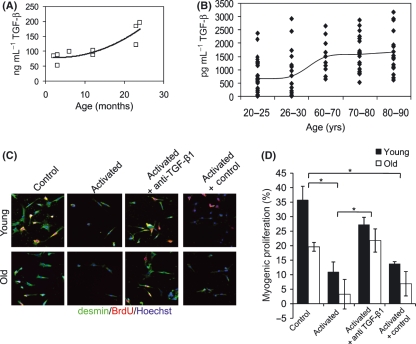
Transforming growth factor (TGF)-β1 levels become elevated in old sera. (A) Mouse sera TGF-β1 becomes elevated with age. Mean serum levels, ±SEM, of TGF-β1 in young (Ym) or old (Om, *n* = 12 for each) mice as determined by sandwich ELISA; *P* < 0.0001. (B) Human TGF-β1 serum levels become elevated with age. TGF-β1 levels in the serum of aged humans (AG: 65–90 years old) vs. young (YG: 20–35 year old) were determined by ELISA. Error bars (SEM) represent the mean of 53 different subjects in each group; *P* < 0.0001. Similar age-related elevations in TGF-β1 were detected by bioactivity assay (Fig. S3). Dot plots represent separate animals (A) and separate individuals (D) of indicated ages. (C) Young and old myofiber explants were isolated at 3 days postinjury, and cultured overnight in Opti-MEM containing either young or old serum alone (control), or serum that had undergone activation of total TGF-β alone, in combination with neutralizing antibody to TGF-β1, or mixed with control serum. BrdU was added for the last 2 h to measure proliferation. Cells were fixed and immunostained for desmin (green) and BrdU (red), with Hoechst (blue) marking all nuclei (as shown in C, quantified in D). Cells were scored in multiple random fields and results are displayed as the mean percent of Desmin^+^/BrdU^+^ cells, ±SD. *P < 0.05 for activated compared to control, activated + antibody compared to activated, and control compared to activate + control. *n* = 3.

To further substantiate these conclusions, we correlated the inhibitory range of TGF-β1 with its levels found in young vs. aged sera. Specifically, we analyzed TGF-β1 levels as a function of age in mice and in humans (Fig. 2A,B). In mice, TGF-β1 levels sharply increased between 12 months (early postreproductive age, analogous to 5th–6th decades in humans) and 24 months (analogous to 8th–9th decades in humans), Fig. 2A. Moreover, this systemic age-related rise was found to be conserved in humans (Fig. 2B), where TGF-β1 plateaus at its highest systemic levels between the 6th and 9th decades of life (i.e. roughly at the onset and progression of age-imposed regenerative decline). Sera TGF-β1 for both species thus becomes elevated from the end of the reproductive period, to the end of lifespan.

Curiously, TGF-β1 levels in young mouse sera (∼100 ng mL^−1^) are predicted to be inhibitory to satellite cell responses (data shown in Fig. 1), even if used at 5–10%, *in vitro*. Therefore, despite apparent pro-myogenic differences between young and old sera, physiological TGF-β1 levels alone do not explain the inhibitory affects of aged sera on myogenic responses. To determine whether these inhibitory affects correlated with biologically active TGF-β1 levels, a bio-activity assay was performed on nonactivated serum samples, using the plasminogen activator inhibitor-1 (PAI-1) promoter luciferase-reporter (Danielpour *et al.*, 1989; Abe *et al.*, 1994), and by ELISA (Supporting Fig. S3C,D). These data clearly demonstrated that while bioactive TGF-β1 was higher in old mouse serum, compared to young, the levels in either sera were minute (> 4 ng mL^−1^) and unlikely to be inhibitory in the 5–10% range examined (Figs S3C,D and 1).

Based on published literature, and in agreement with our results, sera and plasma contain little-to-no active TGF-β1. However, the total TGF-β1 released by platelets during sera collection becomes activated (e.g. by cells in culture), evoking an interplay between positive and negative regulators that determines the intracellular signaling strength. The platelet source of TGF-β1 in our serum samples was corroborated, as expected (Supporting Fig. S3E,F). We further postulated that, in the case of young sera, TGF-β1 activity is attenuated and may therefore render young sera pro-myogenic. To test this, we compared satellite cell myogenic responses in cultures containing serum with (i) endogenous TGF-β activity, (ii) forced-activated endogenous TGF-β and (iii) forced-activated endogenous TGF-β, with subsequent TGF-β1-specific antibody neutralization. As shown in Fig. 2C,D, young serum became inhibitory to satellite cell responses (and similar in this regard to old sera) when endogenous young levels of TGF-β were forced-activated. Importantly, inhibition of myogenic activity was dependent on TGF-β1 and not related to sera-activation, as TGF-β1-specific neutralization restored myogenicity to these cultures. Additionally, when mixed with standard young serum, forced-activated young serum was dominant in myogenic inhibition – suggesting that the presence of negative regulators, rather than the removal of positive regulators, accounts for such affects. These data uncover that the strong inhibitory influence of aged sera (as opposed to the pro-myogenic properties of young sera) are not solely explained by age-related differences in TGF-β1 levels.

### Correlation between systemic levels and myogenic effects of Wnt

Using a luciferase-reporter assay (Kaykas *et al.*, 2004; Brack *et al.*, 2007), we also measured the systemic levels of biologically active Wnt, since an age-dependent increase of circulating Wnt (from ∼10 ng mL^−1^ in young to ∼20 ng mL^−1^ in old sera) was recently reported (Brack *et al.*, 2007). In our experiments, Wnt was undetectable in young and old serum from both humans and mice (Fig. 3A,B). Measurable levels of Wnt in all samples were substantially < 5 ng mL^−1^ (based on a recombinant Wnt standard curve). Interestingly, the relative luciferase activity values were actually higher for young mouse serum, compared to old (Fig. 3B).

**Fig. 4:**
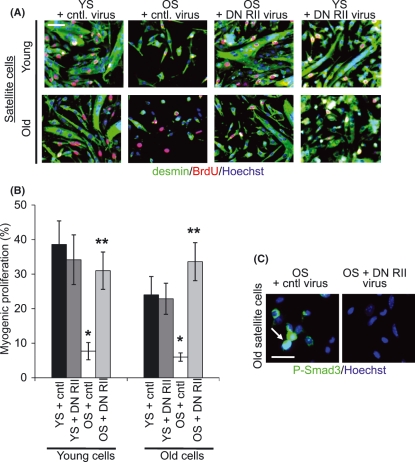
Down-modulation of transforming growth factor (TGF)-β signaling intensity by dominant negative TGF-β RII attenuates old sera-imposed satellite cell regenerative potential inhibition. (A) Young and old mice were injured with CTX, as described; bulk myofibers with activated satellite cells were then explanted and transiently transduced with a TGF-β RII DN-expressing lentivirus, or control virus, *in vitro*. Following transduction, myofiber-derived cells were cultured for 48 h in OPTI-MEM + 5% young (YS) or old (OS) mouse serum. Cells were fixed and analyzed by immunofluorescence for the expression of desmin (green), and levels of BrdU incorporation (red). Hoechst (blue) labels all nuclei. The successful down-modulation of TGF-β signaling by DN RII virus provided restoration of satellite cell regenerative potential in cells exposed to old serum, as evidenced by large number of *de-novo* proliferating desmin^+^ myoblasts that form multinucleated myotubes. Bar = 50 um. (B) Quantification of regenerative potential shown in A, The percent of desmin+/BrdU+ cells is shown as means with standard deviations. (*) indicates *P* < 0.05, compared to treatment with young serum and control virus (Y serum + cntl.) (**) indicates *P* < 0.05, as compared to treatment with old serum and control virus (O serum + cntl.). (C) Levels of nuclear P-Smad3 were diminished in old satellite cells transduced with DN RII-expressing lentivirus, *in vitro*. Old satellite cells, exposed to old serum (OS) plus control virus, or old serum plus DN RII-expressing virus (as indicated), were immunostained for P-Smad3 (green). Hoechst (blue) labels nuclei. As expected, levels of satellite cell nuclear P-Smad3 were diminished in cells transduced with DN RII-expressing virus, as compared to control (white arrow). Bar = 25 μm.

A rescue of satellite cell responses, in the presence of old serum, was reported to be achieved by the inhibition of Wnt via FRPs. In addition, exogenous Wnt3A was reported to inhibit satellite cell myogenic responses, in favor of fibroblast trans-differentiation (Brack *et al.*, 2007). We thus investigated whether Wnt was potentially synergistic with the TGF-β1-dependent attenuation of myogenic potential, and if Wnt sera activity might be detected using satellite cell myogenicity assays. To do so, the myogenic potential of young and old satellite cells was examined in the presence of (i) young serum with exogenous Wnt3A (by itself, or simultaneously with exogenous TGF-β1), or (ii) old serum with exogenous FRP3 (by itself, or simultaneously with TGF-β neutralization). As shown in Fig. 3C–F, exogenous FRP inhibition of Wnt did not rescue myogenic responses in old serum. Moreover, exogenous Wnt3A did not reduce myogenic potential in young serum. Consistent with a reported pro-myogenic activity of Wnt (Brack *et al.*, 2008), Wnt3A also enhanced myotube formation (Supporting Fig. S4A). However, in contrast to the induction of a fibroblastic cell fate (Brack *et al.*, 2007), the presence of nonmyogenic fibroblastic cells (e.g. desmin^−^/BrdU^+^) was not increased, but reduced in the presence of exogenous Wnt3A, thereby further validating the pro-myogenic activity of Wnt. Wnt modulation also failed to synergize with the TGF-β1-promoted inhibition of myogenic responses. In contrast, Wnt3A antagonized TGF-β1 and enhanced the generation of satellite cell-derived, fusion-competent proliferating myoblasts (Fig. 4C–F). Additionally, young satellite cells cultured with young serum had less active GSK3β and more inactive GSK3β (compared to old satellite cells cultured with old serum), suggesting that Wnt signaling is stronger in young conditions (Supporting Fig. S4B,C). These findings were also examined and confirmed using single myofiber assays (Brack *et al.*, 2007 and Supporting Fig. S5). Combined, these results further clarify the respective roles of Wnt and TGF-β1 in adult myogenesis, demonstrate that Wnt activity is not present in sera (determined by bioactivity and satellite cell myogenicity assays), and also demonstrate that Wnt does not contribute to the TGF-β1-dependent inhibition of satellite cell myogenic potential.

### Expression of a dominant-negative TGF-β receptor restores productive myogenic responses to satellite cells exposed to aged serum

After justifying our experimental focus on TGF-β1 as a myogenic inhibitor, we decided to confirm that (i) young sera, compared to old, down-modulates TGF-β1 signaling in satellite cells, and that (ii) the attenuation of TGF-β receptor engagement is sufficient to restore myogenic responses in old cultures. TGF-β receptor levels in satellite cells increase approximately three to fourfold with age (Supporting Fig. S6A), suggesting a compounding effect on signaling from an age-dependent elevation of ligand (Carlson *et al.*, 2008) and receptor. In this regard, we compared the intensity of TGF-β signaling, and the efficiency of myogenic responses, in satellite cells expressing a TGF-β dominant-negative receptor II (TGF-β DN RII), compared to control vector (Tang *et al.*, 1999; Yu & Schaffer, 2006). Young and old satellite cells were activated by muscle injury, followed by progenitor cell isolation and retroviral transduction with TGF-β DN RII or control vector, *ex vivo*. The dominant-negative TGF-β receptor is an inactive kinase, which effectively inhibits signaling by active TGF-β ligands (Tang *et al.*, 1999). As shown in Figs 4A,B and S6B, while having no effect on satellite cells cultured with young serum, TGF-β DN RII expression rescued myogenic proliferation and differentiation in the presence of old serum. The age-related differences in TGF-β signaling strength, and the attenuation of TGF-β signaling DN RII expression, were confirmed by nuclear P-Smad3 analysis in muscle cells (Fig. 4C). Ectopic expression of truncated TGF-β DN RII was confirmed by Western blot (Supporting Fig. S6C).

### Systemic delivery of a TGF-β receptor inhibitor (but not anti-TGF-β antibody or soluble TGF-β receptor) restores old muscle repair, *in vivo*

**Fig. 5:**
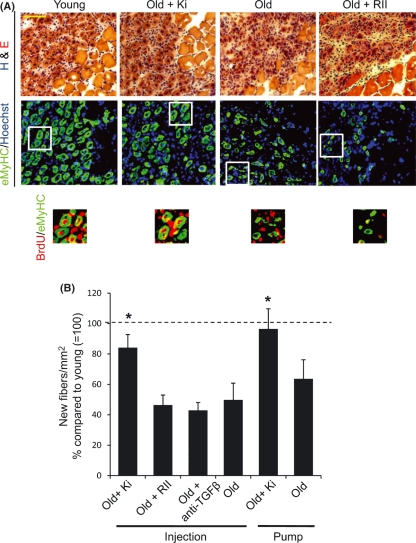
Systemic pharmacological intervention lowers sera transforming growth factor (TGF)-β levels and improves regeneration in old animals. Old or young mice were injected (subcutaneous) with a small molecule inhibitor of the TGF-β RI kinase (Ki), for 2 weeks. Five days before the end of treatment, muscle was injured as described. At the end of treatment, animals were sacrificed and muscle was collected. BrdU was injected (intraperitoneal) at 3 days postinjury to label proliferating, fusion-competent myoblasts. (A) Cryosections (10 μm) were performed in young and old muscle (receiving vehicle alone), old + Ki and old + RII (extracellular portion of TGF-β receptor II). Sections were analyzed by hematoxylin/eosin (H&E) staining and immunostaining for both embryonic myosin heavy chain (eMyHC, shown in green) and BrdU incorporation (shown in red). Hoechst stains nuclei (blue). As shown, the regenerative outcome of old and old + RII was worse than young (judged by scar tissue formation, *de novo* myofiber size/scarcity and reduction of BrdU^+^ nuclei within eMyHC fibers). Scale bar = 100 μm. In contrast, muscle repair was improved by treatment with TGF-β RI kinase (Ki) small molecule inhibitor (similar to young with respect to large/dense eMyHC^+^ myofibers with centrally located BrdU^+^ nuclei). (B) Regeneration was quantified from muscle sections, and is presented as mean percent of newly regenerated myofibers per square millimeter of injury site. Error bars indicate SD, *n* = 3, *P = 0.01 between young or old +Ki, and old.

Examination of our data suggested that sera-derived TGF-β1 was released by platelets during collection procedures. Nevertheless, it remained possible that plasma-based endocrine TGF-β1 could play a role in the inhibition of satellite cells. To examine potential age-dependent endocrine affects of TGF-β1, and to distinguish them from its local influences, we sought to rejuvenate aged muscle repair by attenuating the TGF-β pathway through systemic pharmacological intervention, *in vivo*. Three independent attenuators were used in these experiments: (i) a small molecule inhibitor of TGF-β RI kinase (Singh *et al.*, 2003; Gellibert *et al.*, 2004), (ii) TGF-β neutralizing antibody and (iii) a decoy composed of the extracellular portion of TGF-β receptor II. These molecules were systemically administered for 10 days, followed by cardiotoxin (CTX) muscle injury. Samples were harvested at 5 days postinjury, and examined histologically. The efficiency of adult myogenesis, and age-related changes in this process, were reliably determined by the detection and quantification of newly formed myofibers. Such *de novo* fibers are characteristically small, express eMyHC and have centrally located BrdU^+^ nuclei – indicating that these cells were recently generated by proliferating, fusion-competent myoblasts (Wagers & Conboy, 2005; Carlson & Conboy, 2007a; Carlson *et al.*, 2008). Newly formed muscle fibers are typically numerous and visible in young regenerating muscle, but less numerous in old muscle, which instead shows more mononucleated cells and scar formation (Fig. 5A).

Muscle regeneration from mice treated by systemic administration of the TGF-β RI kinase inhibitor was significantly improved, and similar to the regeneration seen in young control animals (Fig. 5A,B), or young animals receiving TGF-β RI kinase inhibitor treatment (Supporting Fig. S7A–D). In contrast, systemic administration of either TGF-β neutralizing antibody, or soluble TGF-β receptor II, failed to improve myogenic regenerative capacity in old mice (Fig. 5A,B). To control for the biological activity of the anti-TGF-β neutralizing antibody, we performed intra-muscular injections [which enhanced old muscle repair as expected (Supporting Fig. S7E,F)]. In agreement with the data shown in Figs 3 and S5, experimental attenuation of Wnt (by FRP) did not improve the regeneration of old muscle, *in vivo.* Further, Wnt attenuation did not synergize with intra-muscular attenuation of TGF-β, which alone sufficed for the rescue of old muscle repair (Supporting Fig. S7E,F).

**Fig. 6:**
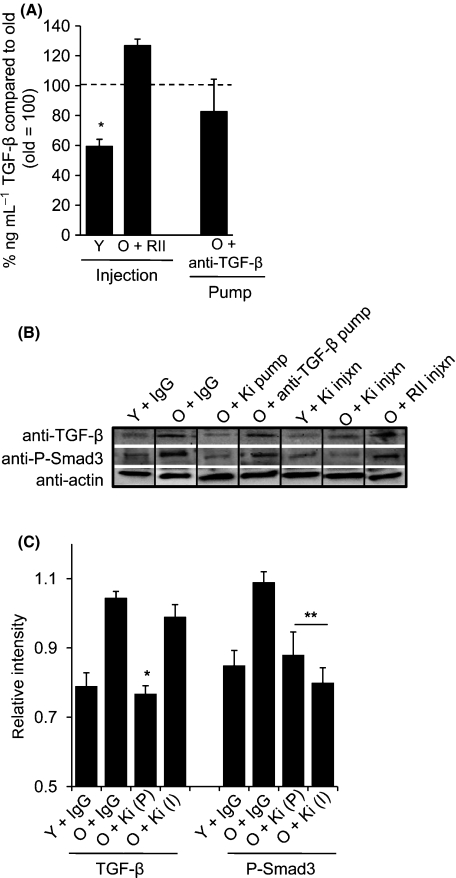
Systemic pharmacological intervention lowers sera transforming growth factor (TGF)-β levels and improves regeneration in old animals. (A) Myofibers were isolated from young, old and old mice with indicated treatments for TGF-β systemic down-modulation. Cell lysates were then analyzed by Western blot for levels of secretory TGF-β and P-Smad3 signaling strength. Actin immunodetection was used as a loading control. As expected, higher levels of TGF-β and P-Smad3 were detected in old samples, as compared to young. O + Ki pump showed reduction in both TGF-β and P-Smad3, whereas O + Ki injection only reduced P-Smad3 signaling strength. (B) Results of multiple Western blot assays were quantified (normalization of TGF-β and P-Smad3 pixel density by actin-specific pixel density) and are depicted by relative pixel intensity, as shown; (*) indicates *P* < 0.01, O Ki compared to old and young. Error bars indicate SD. *n* = 3–4. (C) TGF-β levels in serum for each animal in young, old or old + experimental treatments were determined by ELISA. Shown are mean values with standard deviations (*n* = 3–12, *P* ≤ 0.05 between O + Ki pump and O + anti-TGFβ pump, and O + Ki/O + RII injection.

To control for potential half-life differences of the anti-TGF-β neutralizing antibody, compared to TGF-β RI kinase inhibitor, we also employed osmotic subcutaneous pumps for the constitutive systemic delivery of these molecules. Under these conditions, the anti-TGF-β neutralizing antibody still failed to significantly enhance repair of old muscle, while TGF-β RI kinase inhibitor robustly promoted muscle repair (Fig. 5B). These key data suggest that endocrine TGF-β1 does not play a major role in the age-related decline of muscle regeneration, *in vivo*. Furthermore, ELISA assays on systemic TGF-β1 levels not only corroborated, but strengthened this conclusion (Fig. 6A). These data demonstrated that TGF-β1 neutralizing antibody, or soluble TGF-β receptor II, failed to reduce circulating levels of TGF-β1 in old mice (Fig. 6A). In agreement with other reports, our findings suggest that bio-active TGF-β1 is not present in the circulation, which would be recognizable by the herein tested antibody or cognate receptor (Annes *et al.*, 2003; Hyytiainen *et al.*, 2004; Feng & Derynck, 2005).

The rejuvenating effects produced by TGF-β RI kinase inhibitor systemic delivery are best explained by the attenuation of local TGF-β signaling in all tissues, including skeletal muscle. As shown in Fig. 6B,C, this hypothesis is directly substantiated by our experimental data. Namely, the high levels of P-Smad3 and TGF-β1, typical of old muscle (Carlson *et al.*, 2008), are significantly reduced in old mice receiving systemically administered TGF-β RI kinase inhibitor, but not in old mice receiving anti-TGF-β antibody or soluble receptor. Importantly, osmotic pump delivery TGF-β RI kinase inhibitor not only reduced P-Smad3 levels, but also diminished levels of TGF-β1 production by muscle cells, suggesting positive feed-back regulation in the TGF-β pathway (Fig. 6B,C).

Together, these data establish that systemically administered TGF-β RI kinase inhibitor acts locally to attenuate TGF-β1/P-Smad3 signaling in satellite cells, and restores muscle repair, *in vivo*. These findings are also in agreement with our previous report (Carlson *et al.*, 2008). In contrast, systemic levels and intra-muscular TGF-β1 signaling strength are not down-modulated, and old muscle repair is not enhanced by circulating neutralizing antibody or soluble receptor (which do not access endocrine or tissue TGF-β when administered systemically). Thus, while TGF-β1 is released by platelets during sera collection, and does exhibit an inhibitory and age-dependent influence on satellite cells, *in vitro* (Figs 1, 2 and S2), there is no evidence for TGF-β1 endocrine anti-myogenic activity, *in vivo*.

## Discussion

The data presented in this work establish the anti-regenerative properties and inhibitory range of TGF-β1. These data also demonstrate that the age-related increase in TGF-β1 levels is conserved between mouse and human sera (Figs 1 and 2), and may therefore be universal to the aging process of mammals. In our studies, there was less variation in measurable TGF-β1 levels within mice (when compared with humans), likely owing to their environmental and genetic homogeneity. It is also important to note that our reported human TGF-β1 levels are representative of the healthy aging population, as individuals for these studies were prescreened for confounding medical conditions. Despite this, none of the aged individuals (≥ 60 years) had less than measurable levels of TGF-β1, as opposed to many of the young individuals (≤ 30 years) enrolled in these studies. Considering that TGF-β1 is broadly produced, and signals to a variety of cells, the age-related and evolutionarily conserved elevation of its functional levels in old sera may affect stem cell responses in various other tissues. Such a finding may provide a preliminary explanation for the general organ stem cell regenerative potential decline of mice and humans.

With respect to satellite cell activation, TGF-β1 operates in a threshold fashion (Fig. 1), and appears to function through the TGF-β II receptor and P-Smad signaling (Figs 4 and 6). Our findings suggest that certain levels of TGF-β1 are actually required for normal satellite cell responses (Fig. 1). Experimental down-modulation of this pathway is therefore expected to be typically harmful, unless it is transient (Figs 1, 4––6) or performed to precise ‘beneficial’ levels. These conclusions fit well with studies, reporting that among other important functions, TGF-β1 signaling is needed for efficient immune cell and wound clearance responses (Dunker & Krieglstein, 2000). TGF-β proteins have been shown to behave as morphogens which, depending on concentration, induce various gene subsets and promote different effects (McPherron *et al.*, 1997; Itoh *et al.*, 2003; Kamanga-Sollo *et al.*, 2003; Muller *et al.*, 2003; Wharton *et al.*, 2004). The morphogenic nature of the TGF-β family agrees with our conclusion that, at specific concentrations, TGF-β1 is permissive or nonpermissive to productive myogenic regenerative responses (Figs 1, 2 and S1, S2). Consequently, pronounced down-modulation of TGF-β1 causes immune disorders, inflammation and organ dysfunction. This complicates the use of TGF-β1 attenuators, such as antibodies and small molecules (delivered by pumps, or otherwise), or the use of genetically altered mice, which do not survive into old age if they harbor TGF-β pathway insufficiencies. For example, genetic deficiencies in TGF-β1,2,3, TGF-β RI,II,III, Smad2, Smad4 and others (Oshima *et al.*, 1996; Bonyadi *et al.*, 1997; Larsson *et al.*, 2001; Brionne *et al.*, 2003; Andrews *et al.*, 2006) all cause embryonic or neonatal lethality, due to the abnormal development of various organ systems. Furthermore, Smad3 knockout animals survive after birth, but die before old age due to chronic infections, accelerated rate of cancers and T-cell dysfunction (Datto *et al.*, 1999; Yang *et al.*, 1999). TGF-β1 heterozygous mice also survive after birth, but have increased neuronal abnormalities, inflammation and fibrosis of blood vessels (Wyss-Coray *et al.*, 1997, 2001; Brionne *et al.*, 2003). Combined, these data agree with our conclusion that prolonged, or sustained, experimental down-modulation of TGF-β1 levels will perturb cellular function.

Our work reveals that platelet-derived sera TGF-β1 levels, or endocrine TGF-β1 levels, do not explain the age-dependent inhibition of tissue regeneration by this cytokine. Specifically, if all endogenous young sera TGF-β1 were active, it would readily suppress satellite cell responses, *in vitro* (Figs 1 and 2). Further, neutralization of circulatory TGF-β1 is not achievable by experimental ligand traps, *in vivo* (Figs 5 and 6). These data suggest the presence of a natural TGF-β1 modifier/decoy (predictably more active in the young), which is likely endocrine in nature, or released during platelet activation (Figs 1 and 2). Future work on the characterization of such natural systemic antagonist(s) of TGF-β1 may also explain the regenerative phenotypes observed in hetereochronic parabiosis (Conboy *et al.*, 2005).

This work further confirms and extrapolates the autocrine/paracrine mode of TGF-β age-dependent inhibition of organ stem cell responses (Carlson *et al.*, 2008). Specifically, old tissue repair was improved only when P-Smad3 levels were attenuated locally in muscle (e.g. through small molecule systemic delivery), which is also expected to attenuate TGF-β signaling broadly in all tissues (Figs 5 and 6). In contrast, systemically administered neutralizing antibody, or soluble receptor, failed to restore old muscle repair. These molecules did not attenuate endocrine or local TGF-β1, nor diminish P-Smad3 levels in satellite cells, *in vivo* (Figs 5 and 6). These data also predict the presence of a positive feed-back loop, where a certain TGF-β signaling strength is required to maintain TGF-β1 ligand expression (Fig. 6), which was additionally suggested by TGF-β level reduction in muscle treated with RNAi to Smad3 (Carlson *et al.*, 2008). It remains to be determined whether there is a broad TGF-β1 increase within old tissues (in addition to skeletal muscle fibers), or if a narrow subset of cells in aged organisms overproduce TGF-β1.

While the involvement of additional TGF-β family members (or other age-dependent cytokines involved with muscle repair decline) is certainly not ruled out, TGF-β1 was identified here as a key, age-related inhibitor of myogenic responses. Additionally, myostatin was previously reported to not locally increase in old muscle tissue (Carlson *et al.*, 2008), thus implicating the ubiquitous TGF-β1, rather than tissue-specific family members in these age-related phenotypes. While other circulatory molecules may contribute to the aging of muscle stem cell responses, our data suggest that Wnt is not a systemic age-dependent attenuator of myogenicity (Figs 2, 3 S2 and S4). These data are in apparent disagreement with those reported by Brack *et al.* (2008); and further study will resolve whether Wnt is present in the circulation, is upregulated with aging and becomes inhibitory to myogenic responses at some age-specific level. Of note, in reports examining Wnt in young mice only, it is suggested that Wnt promotes satellite cell myogenicity for both differentiation of myoblasts to myotubes, and with respect to satellite cell self-renewal (Brack *et al.*, 2008; Le Grand *et al.*, 2009).

Comprehensively, these studies identify physiological sources of TGF-β1 that are responsible for age-related muscle repair inhibition, and point toward novel therapeutic strategies for the enhancement of organ regenerative potential. In proof of principle research, intra-muscular attenuation of Smad3, by RNAi, rescued aged muscle repair (Carlson *et al.*, 2008). Such methodology, however, is not easily applicable to an organism-wide restoration of old organ repair. In contrast, our herein reported systemic delivery of a TGF-β receptor kinase inhibitor allows one to reach all tissues, to recalibrate TGF-β1 ligand levels to their youthful states and to potentially rejuvenate their regenerative function.

## Experimental procedures

### Cell culture

Myofiber explant cultures and primary myogenic progenitor cells were generated from C57/Bl6 mice as described previously (Conboy *et al.*, 2003). Myoblasts were maintained in growth media (Ham’s F-10, 20% FBS, 5 ng mL^−1^ basic-FGF and penicillin–streptomycin). Opti-MEM was used to culture myofiber explants in 10% young, 10% old or 10% young plus old mouse serum (5% each). Differentiation media consisted of DMEM and 5% horse sera. Basic-FGF was obtained from Sigma (St Louis, MO, USA) and R&D systems (Minneapolis, MN, USA), Opti-MEM was obtained from Invitrogen (Carlsbad, CA, USA), and all other cell culture reagents were obtained from Cellgro (Herndon, VA, USA).

### Retrovirus production and transfection

pMSCV-TβRIIDN retroviral vector was a kind gift from Dr Chung Lee (Kundu *et al.*, 2000; Shah *et al.*, 2002). TβRIIDN was excised from the vector using *Eco*RI/*Bam*HI, blunted with Klenow (NEB) and inserted into the MoMLV retroviral vector pCLPCGFP (Ignowski & Schaffer, 2004). pCLPCGFP was first digested with *Bam*HI, following which it was also blunted with Klenow and ligated with the TβRIIDN fragment. The resulting pCLPC TβRIIDN vector was sequenced to confirm its sequence and directionality of insertion. A plasmid made by ligating the blunt ends of the pCLPC vector was used as a control. To produce retrovirus, HEK 293T cells (cultured in Iscove’s modified Dulbecco’s medium (Hyclone, Logan, UT, USA) with 10% fetal bovine serum (Invitrogen) and 1% penicillin–streptomycin (Gibco, Carlsbad, CA, USA) and maintained at 37 °C and 5% CO_2_) were co-transfected with the retroviral vector (pCLPC TβRIIDN or control plasmid) and the helper plasmids pCMV-VSVG, a vector encoding the viral envelope protein from the vesicular stomatitis virus and pCMV-gagpol, a vector encoding the enzymatic and structural retroviral proteins, using the calcium phosphate transfection method as described (Yu & Schaffer, 2006). Medium was changed 12 hours after transfection. Forty-eight hours after transfection, the cell culture supernatant, containing the virus, was collected and purified by ultra centrifugation through 20% sucrose in phosphate-buffered saline (PBS) and resuspended in PBS. The virus also encoded a gene that rendered it resistant against puromycin, and was titered on 293T cells using WST1 reagent (Roche, Mannheim, Germany) following the manufacturer’s protocol.

Viral transduction was performed on bulk myofiber explants with activated satellite cells, isolated 3 days postinjury from CTX-injured young and old animals. Cells were infected with TβRIIDN (2 × 10^6^ TU mL^−1^), or control viruses (1 ×10^7^TU mL^−1^), at assay-dependent MOIs, and cultured for 48 hours in the presence of OPTI-MEM, +5% young or old mouse serum prior to fixation and analysis. No tetracycline was added in these experiments.

### Animal strains

Young (2–4 month) and old (20–24 month) C57/Bl6 male mice were obtained from Jackson Laboratories (Bar Harbor, ME, USA) and NIA (Bethesda, MD, USA) respectively.

### Antibodies and reagents

Antibody against embryonic myosin heavy chain (developmental) was purchased from Vector Laboratories (Burlingame, CA, USA) and the Developmental Studies Hybridoma Bank. Phosphorylated-Smad2/3 antibody [1:200 WB (Western blot), 1:100 IF (immunofluorescence) was obtained from Chemicon (Temecula, CA, USA) and Santa Cruz Biotechnologies (Santa Cruz, CA, USA; sc-11769)]. Bioactivity neutralizing antibody against TGF-β1 (ΜΑΒ240), rTGF-β1 (240-B) rWnt3a (1324-WN) sFRP3 (592-FR) and huTGF-β RIII (242-R3) were purchased from R&D Systems. Delta (9120) and TGF-beta receptor I (SC 9048) (1:100 WB) antibodies were obtained from Santa Cruz Biotechnologies. Activated Notch (1:500 WB) (ab8925) and BrdU (1:100 IF) (ab6326) antibody were obtained from Abcam (Cambridge, MA, USA). Desmin (1:25 IF) (clone DE-U-10, #D1033) and actin (1:250 WB, A5060) antibodies were purchased from Sigma.

### Muscle injury

Isoflurane was used to anesthetize the animal during the muscle injury procedure. For bulk myofiber satellite cell activation, tibialis anterior and gastrocnemius muscles were injected with CTX I (Sigma) dissolved at 100 μg mL^−1^ in PBS, at 2–5 sites for each muscle for approximately 50 μL g^−1^ muscle. Muscles were harvested 3 days later. For focal injury, to assay regeneration *in vivo*, 2–3 μL of 1 mg mL^−1^ CTX was injected at one site to the middle of the tibialis anterior, or two sites at the gastrocnemius, and muscle harvested 5 days later. In some experiments, FRP3 (500 ng), anti-TGF-β (10 ng), FRP3/anti-TGF-β or IgG (10 ng) were injected intramuscularly into injury sites, 24 h following initial injury. In these studies, BrdU (50 mg kg^−1^) was injected IP at 2 days following injury.

### Immunofluorescence and histological analysis

All muscle tissue was dissected, flash frozen in OCT compound (Tissue Tek; Sakura Finetek, Zoeterwoude, The Netherlands) and cryo-sectioned at 8–10 μm, as previously described (Conboy *et al.*, 2003). Muscle sections were stained with aqueous hematoxylin and eosin (H&E), as per the manufacturer’s instructions (Sigma-Aldrich). Regeneration and myogenic potential was quantified by examining injury sites from representative sections along the muscle (spanning the volume of injury), then measuring the injured/regenerating area using Adobe Photoshop Elements. In all experiments, regenerating tissue was obtained from both tibialis anterior and gastrocnemius muscles. Cryo-sectioning was performed through the entire volume of muscle (typically 50–70 sections total, done at 200 μm intervals), thereby serially reconstituting the entire tissue, *ex vivo.* Myofiber regeneration was quantified by counting total newly regenerated fibers at injury epicenters, per volume of injury (typically ∼1 mm^2^) and dividing fibers per area. Immunostaining was performed as described (Conboy *et al.*, 2003). Briefly, after permeabilization in PBS + 1% FBS + 0.25% Triton-X-100, tissues and cells were incubated with primary antibodies in staining buffer (PBS + 1% FBS) for 1 h at room temperature, followed by 1 h incubation fluorochrome-labeled secondary antibodies (ALEXA; Molecular Probes, Carlsbad, CA, USA at 1:1000). P-Smad2/3 and BrdU-specific immunostaining required an extra step of 2 m HCl treatment (denaturation of DNA) before permeablization. The index of myogenic potential was evaluated, based on BrdU/eMyHC costaining, as previously described (Carlson & Conboy, 2007a; Carlson *et al.*, 2008).

### Depletion of TGF-β from culture media

Opti-MEM containing either 10% young or old mouse serum was incubated in the presence of 5 μg mL^−1^ TGF-β antibody (R&D Systems) overnight at 4 °C. Protein-G agarose was then added to the media/antibody mix and incubated for 4 h at 4 °C. The mixture was then centrifuged at 10 000 RCF for 5 min. Supernatant was collected and centrifugation was repeated. TGF-β depleted supernatant was used for culture.

### Plasma/platelet preparation and activation of serum TGF-β

For plasma preparation, < 100 μL was collected from the tail into 10 μL of 100 mm EDTA. Red blood cells were removed by sedimentation, twice at 150 *g* for 10 min, followed by platelet sedimentation for 10 min at 1500 *g*. Platelet-poor plasma was re-cleared by an additional sedimentation for 10 min at 1500 *g*. Platelets were washed once with 0.08 m sodium citrate, pH 6/0.9 g L^−1^ sodium chloride, sedimented again at 1500 *g*, and resuspended in cold water before flash freezing. Serum TGF-β was activated by mixing 100 μL of serum with 100 μL 10 m Urea/2.5 m acetic acid, followed by incubation for 10 min at room temperature. Acid treatment was neutralized with 2.7 m sodium hydroxide/1 m HEPES, and salts were removed via ultrafiltration (Vivaspin 500, 3 kDa MWCO; GE Healthcare, Piscataway, NJ, USA).

### Measurement of bioactive levels of TGF-β in serum

Serum was isolated as previously described (Conboy *et al.*, 2003). Briefly, blood cells were coagulated at 37°C for 15 min and centrifuged to isolate serum. In the luciferase-reporter trans-activation assay, the biological activity of TGF-β was measured via induction of the PAI-1 promoter in mink lung epithelial cells stably expressing a PAI promoter-luciferase construct (Abe *et al.*, 1994). Briefly, 3 × 10^4^ transfected cells were plated in 96-well plates in 50 μL of Dulbecco’s modified eagle medium (DMEM) with 0.5% fetal bovine serum and incubated for 3 h at 37 °C in 5% CO_2_. Experimental sample (50 μL of DMEM with young or old mouse serum at 1:2 and 1:10 dilutions) was added to triplicate wells and incubated for 16 h. Cells were then washed with PBS and lysed. The luciferase activity in the lysate was measured in an EG&G Berthold Microlumat LB 96P (Oak Ridge, TN, USA) for 10 s, immediately following auto-injection of 20 μL of luciferase substrate and recorded as relative light units integrated over time. A TGF-β standard curve was performed to determine the total amount of active TGF-β. Neutralizing antibodies specific for individual TGF-β (R&D Systems) were added to demonstrate specificity.

### Measurement of bioactive levels of WNT in serum

L cells, stably harboring SuperTopFlash (Kaykas *et al.*, 2004) and pEF1-LacZ constructs (‘LSL cells’), were plated into 96-well microtiter dishes at approximately 80–90% confluency. After ∼6 h postplating, media was removed and LSL cells were treated with serum or Wnt protein diluted into complete media (DMEM/10% FBS plus antibiotics) for approximately 16 h at 37 °C luciferase activity was measured in a Lumat LB 9507 luminometer (Berthold) using the Dual light TROPIX kit according to manufacturer’s instructions. All assays were performed in triplicate, and the relative luciferase units were normalized to LacZ readings.

### Measurement of serum TGF-β levels

Mouse blood was collected upon euthanasia, and serum was prepared as above. Mouse serum levels were determined by sandwich ELISA, as per the manufacturer’s protocol (R&D Systems **#**DY1679), with the recommended pretreatment of sample with acid/urea, followed by neutralization. To avoid dilution effects on measurement, peak readings calculated from the corrected dilution curve were used for comparison among samples. Serum from aged and young human subjects was collected and stored in aliquots at −70 °C until used. Young donors were between 20 and 35 years of age and elderly donors were between 65 and 90 years of age. Elderly subjects belonged to middle-class socio-economic status, and were living independently. This study was approved by the Institutional Review Board of the University of California, Irvine. Human TGF-β levels were determined using a specific ELISA kit (BD Biosciences, San Jose, CA, USA) with the recommended pretreatment of sample with acid, followed by neutralization with the base.

### Western blot analysis

Western blotting was performed according to standard protocols. Typically, 30 μg of protein extract from myofibers, or satellite cells, were run on SDS–PAGE gels (Bio-Rad, Hercules, CA, USA). Primary antibodies were diluted in 5% nonfat milk in 1× PBS-T at indicated dilutions (see above). HRP-conjugated secondary antibodies (Santa Cruz Biotech) were diluted 1:1000 in 1× PBS, 2%BSA, and 0.5% Tween-20. Nitrocellulose membranes were treated with secondary antibodies for 1 h at room temperature, developed using ECL reagent (Amersham, Sunnyvale, CA, USA), and imaged on a Bio-Rad Chemidoc XRS.

### Pharmacological intervention

For modulating TGF-β levels systemically, the TGF-β RI kinase inhibitor (Calbiochem/EMD Chemicals Gibbstown, NJ, USA; catalog # 616452) was delivered at 10 nmole over 2 weeks, by injecting twice daily for 4 weeks (subcutaneous) with 100 μL of a 1:10 dilution of 3.4 mm inhibitor (dissolved in dimethylsulfoxide), diluted in PBS with diluted DMSO as control. Five days before the end of treatment, muscle was injured as described. At the end of treatment, animals were sacrificed and muscles were collected. BrdU was injected (intraperitoneal) at 3 days postinjury to label proliferating, fusion-competent myoblasts.

### Statistical analysis

A minimum of three replicates were performed for each experimental condition. Quantified data are presented as means and standard deviations. Analysis of variance was used to compare data from different experimental groups. *P-*values of < 0.05 were considered statistically significant.

## Supporting Information

**Fig. S1** Time-course analysis of myogenic proliferation and differentiation.

**Fig. S2** Old serum inhibits myogenic differentiation by TGF-β-dependent mechanism.

**Fig. S3** Old animals have high levels of total platelet and bioactive sera TGF-β.

**Fig. S4** Old serum inhibits myogenic differentiation by a TGF-β-dependent and Wnt-independent mechanism.

**Fig. S5** Wnt does not reduce myogenic responses in young sera and FRP does not rescue myogenic responses in old sera in single myofiber systems.

**Fig. S6** Old animals have high levels of TGF-β receptor expression; dominant negative TGF-β RII is expressed by virally infected muscle stem cells.

**Fig. S7** Systemic pharmacological intervention does not adversely affect TGF-β sera levels and muscle regeneration in young animals; Wnt down-regulation by FRP does not rescue regeneration of old muscle, *in vivo*.

As a service to our authors and readers, this journal provides supporting information supplied by the authors. Such materials are peer-reviewed and may be re-organized for online delivery, but are not copy-edited or typeset. Technical support issues arising from supporting information (other than missing files) should be addressed to the authors.
