# Supplementary figures and images for: Relative roles of TGF-β1 and Wnt in the systemic regulation and aging of satellite cell responses
Source: Aging Cell. 2009 Dec;8(6):676–89. doi: 10.1111/j.1474-9726.2009.00517.x (PMC2783265; doi:10.1111/j.1474-9726.2009.00517.x)

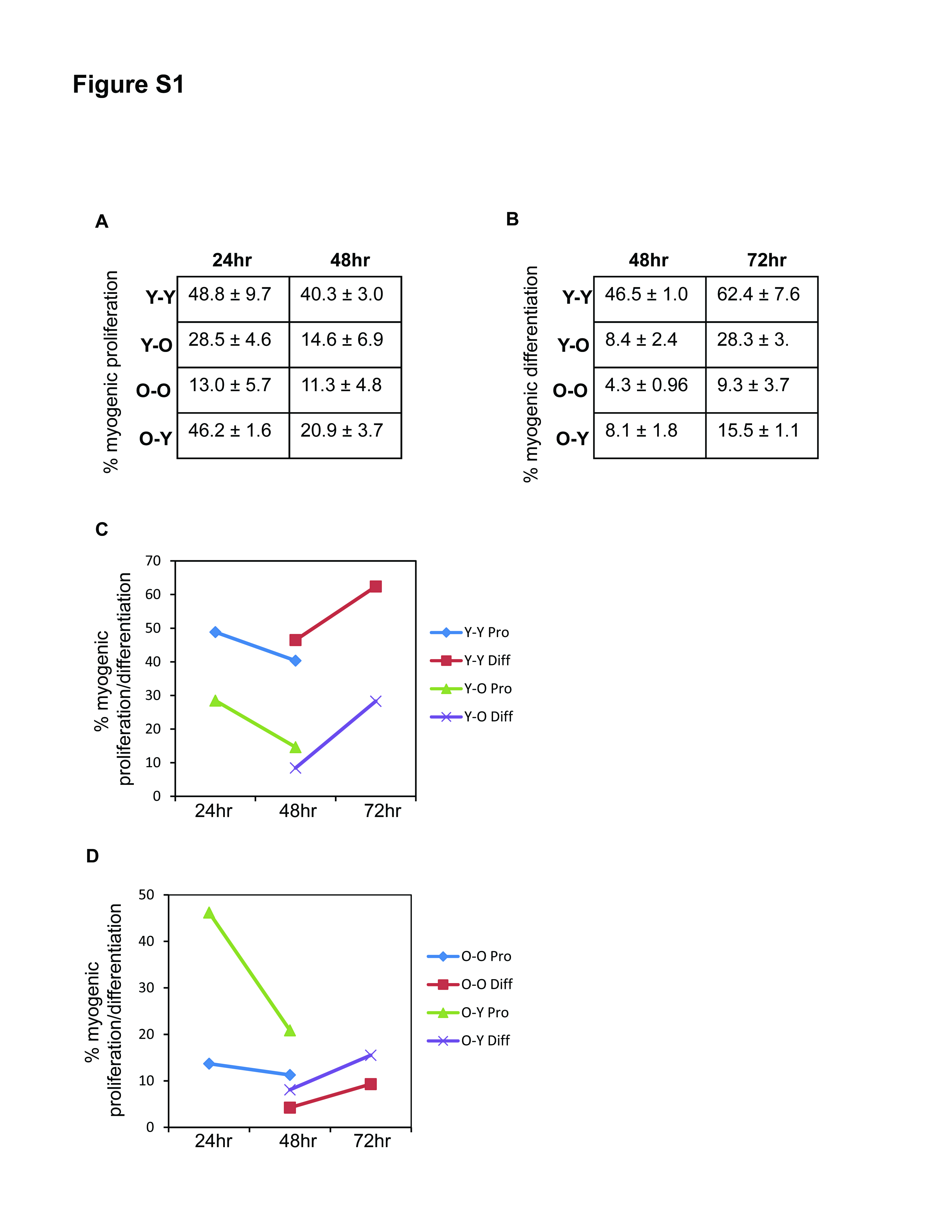

Supplement: Supplementary file 1 [file ace0008-0676-SD1.tif]

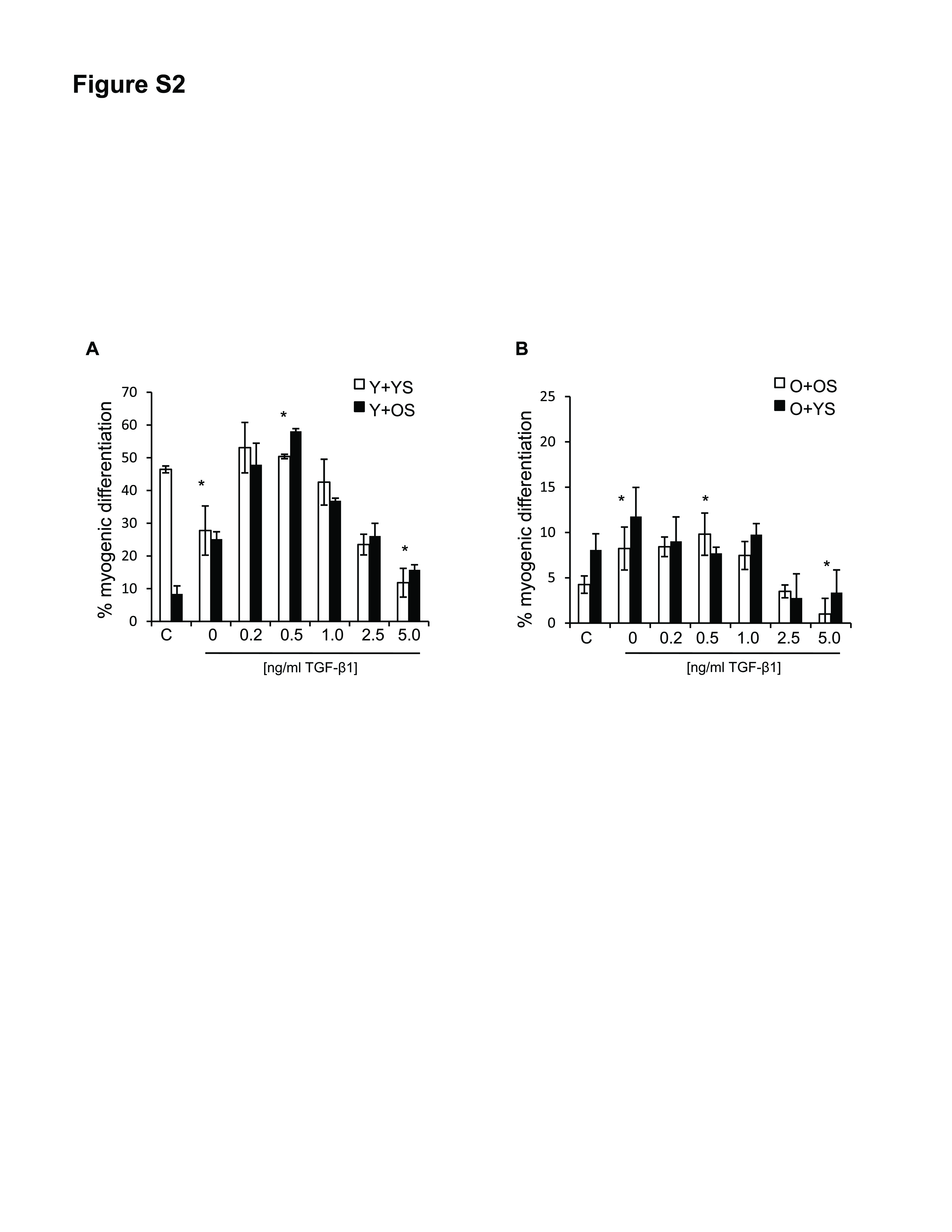

Supplement: Supplementary file 2 [file ace0008-0676-SD2.tif]

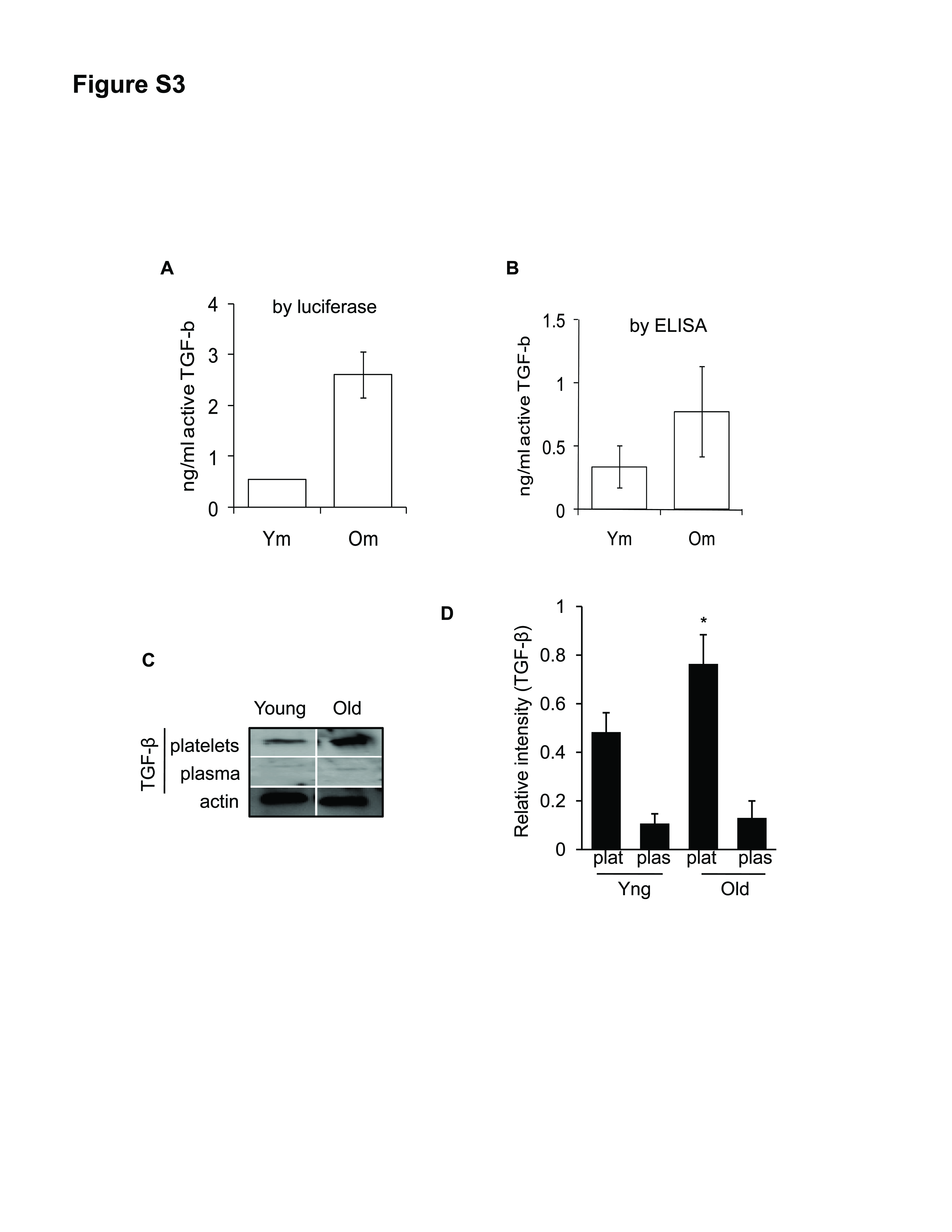

Supplement: Supplementary file 3 [file ace0008-0676-SD3.tif]

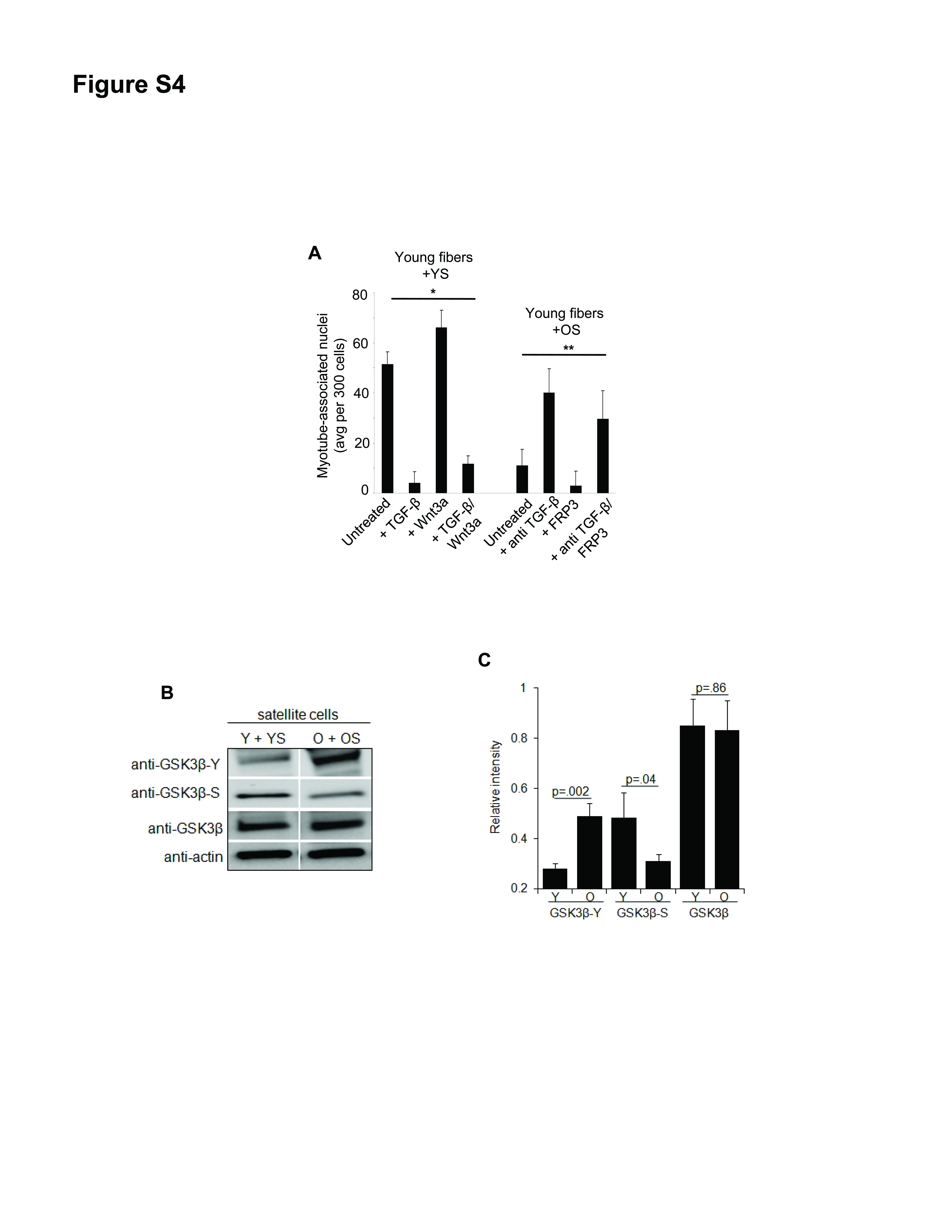

Supplement: Supplementary file 4 [file ace0008-0676-SD4.tif]

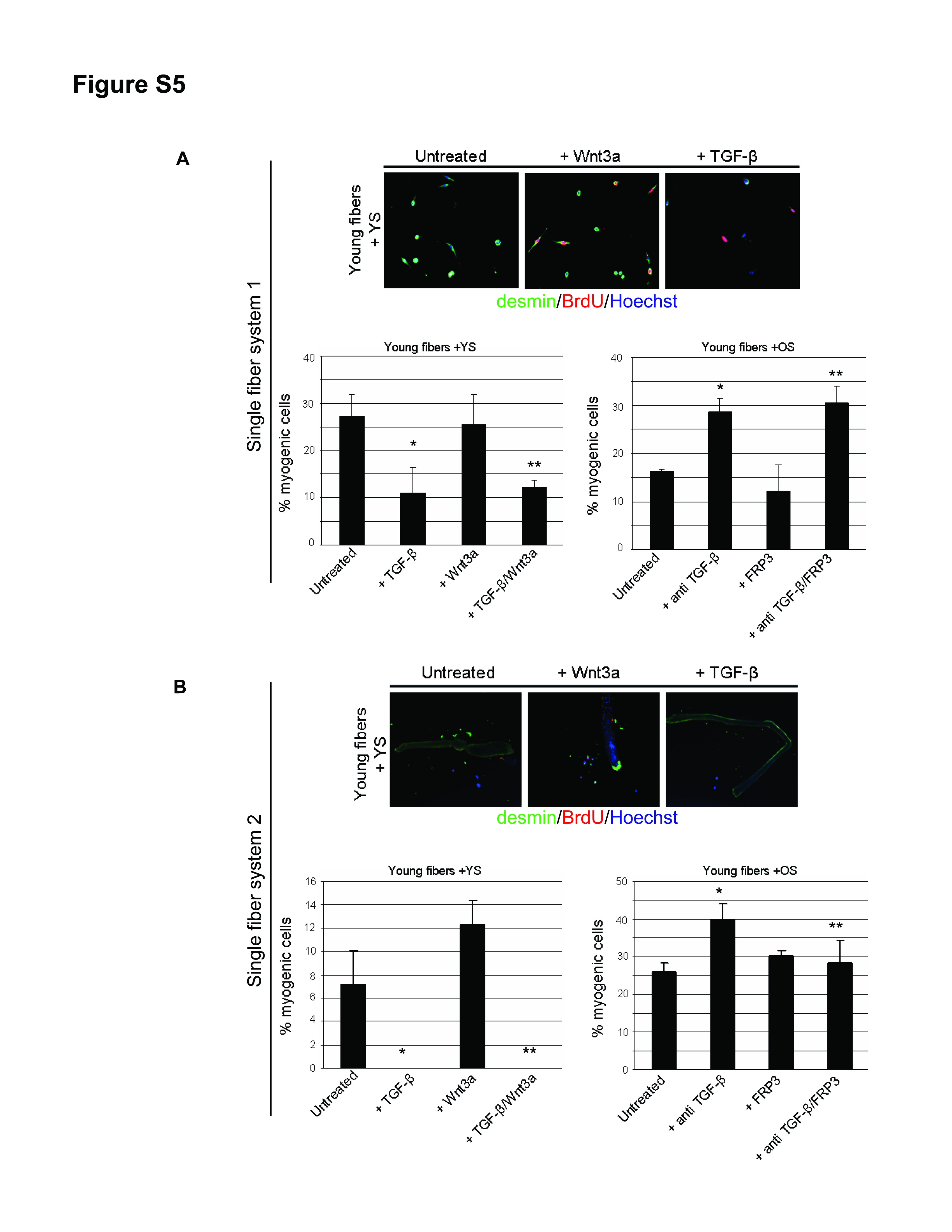

Supplement: Supplementary file 5 [file ace0008-0676-SD5.tif]

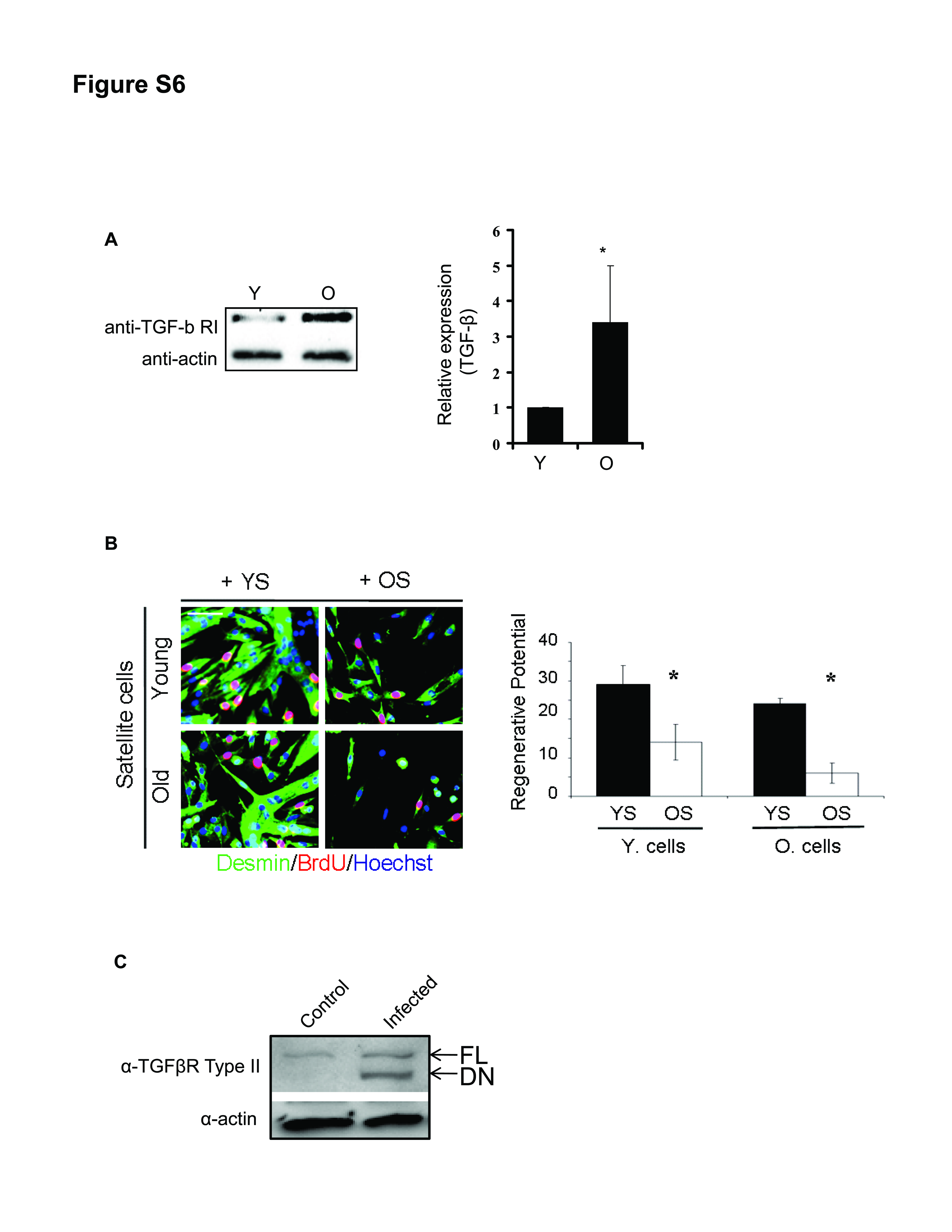

Supplement: Supplementary file 6 [file ace0008-0676-SD6.tif]

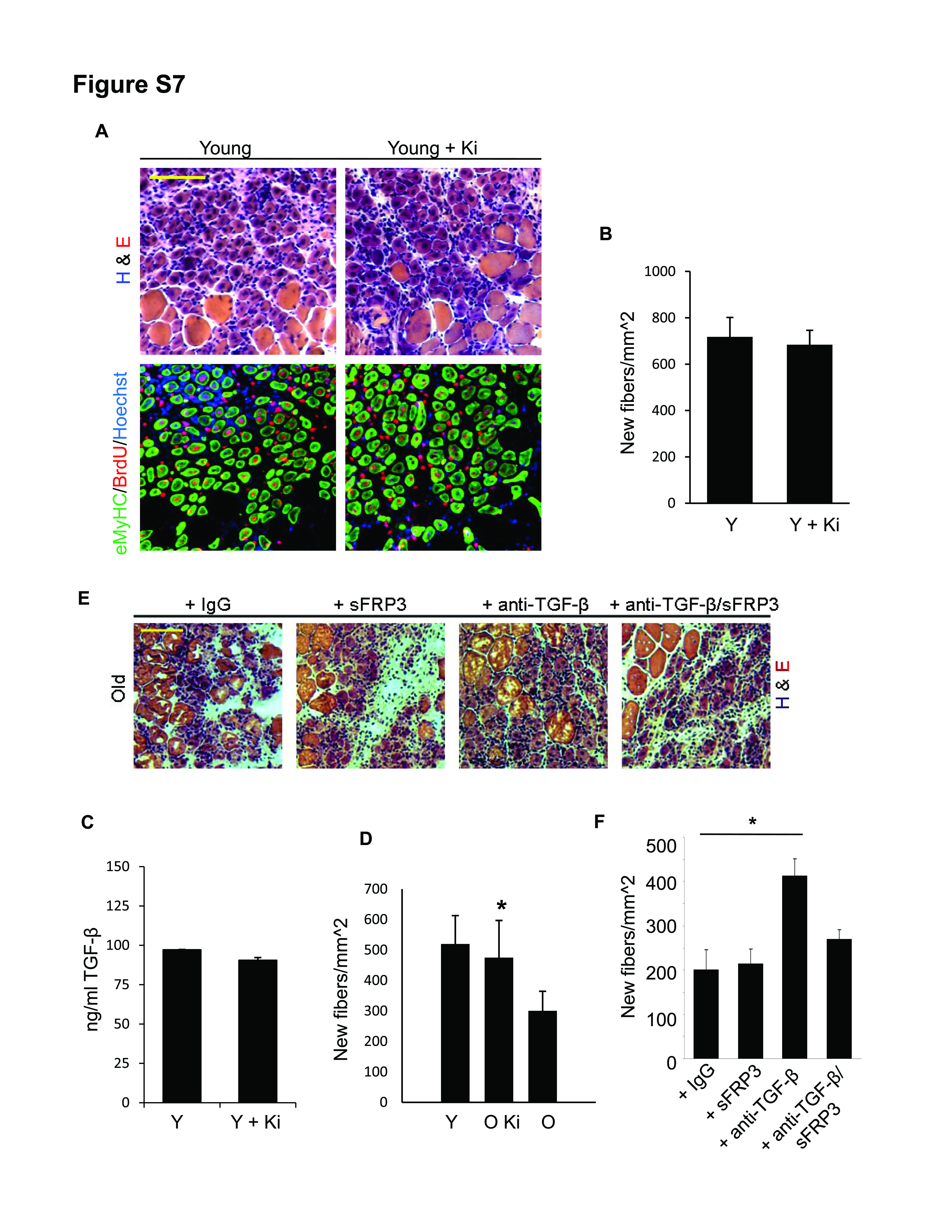

Supplement: Supplementary file 7 [file ace0008-0676-SD7.tif]
